# Multi-Walled Carbon Nanotubes Solid-Phase Extraction and Capillary Electrophoresis Methods for the Analysis of 4-Cyanophenol and 3-Nitrophenol in Water

**DOI:** 10.3390/molecules25173893

**Published:** 2020-08-26

**Authors:** Zeid A. ALOthman, Ahmad Yacine Badjah, Marcello Locatelli, Imran Ali

**Affiliations:** 1 Advanced Materials Research Chair, Chemistry Department, College of Science, King Saud University, P. O. Box 2455, Riyadh 11451, Saudi Arabia; ybadjah@ksu.edu.sa; 2 Department of Pharmacy, University “G. d’Annunzio” of Chieti-Pescara, Build B, level 2, 66100 Chieti, Italy; m.locatelli@unich.it; 3 Department of Chemistry, College of Sciences, Taibah University, Al-Medina Al-Munawara 41477, Saudi Arabia; 4 Department of Chemistry, Jamia Millia Islamia, Jamia Nagar, New Delhi 110025, India

**Keywords:** 4-cyanophenol, 3-nitrophenol, multi-walled carbon nanotubes, water, capillary electrophoresis, solid-phase extraction

## Abstract

Analysis of 4-cyanophenol and 3-nitrophenol was carried out using multi-walled carbon nanotubes-based solid-phase extraction (SPE) and capillary electrophoresis (CE) methods. Capillary electrophoresis was carried out with 18 kV voltage, 214 nm detection, and phosphate buffer (pH 7.0, 15 mM) as background electrolyte at 25 ± 1 °C temperature with 15.05 and 16.5 min migration times of 4-cyanophenol and 3-nitrophenol. The separation and resolution factors were 1.10 and 2.90. The optimized experimental conditions were 40 mg/L concentration, 1.0 g multi-walled carbon nanotubes (MWCNTs) per SPE cartridge, 5.0 mL/min flow rate of water, 0.1 mL flow rate of eluting solvent. The maximum recoveries were 91% and 98% at 0.1 mL/min flow rate of 4-cyanophenol and 3-nitrophenol. These methods were applied successfully for extraction and estimation of 4-cyanophenol and 3-nitrophenol in the municipal wastewater. The reported methods are reproducible, efficient, and practical for the estimation of these phenols in water.

## 1. Introduction

Water quality determination by analyzing the various constituents in water is an important area from the health point of view [1,2]. Water quality is determined by physical, chemical, and biological parameters [3]. The chemical parameters are of utmost importance as these are responsible for determining water quality up to a great extent. The presence of toxic organic contaminants in the water is dangerous for our health as many organics are carcinogenic in nature [4,5]. Phenolic compounds are very toxic to human beings; even at trace levels [6,7,8,9]. The prime source of phenolic contamination in water is industrial and domestics discharges, pesticide use, and automobile exhaust [10,11,12,13]. 4-Cyano- and 3-nitrophenols are found in some industrial effluents and toxic; if the effluent is discharged on to land or mixed in surface water without proper treatment [14,15,16,17,18]. 4-Cyanophenol and 3-nitrophenol are toxic when swallowed. Besides, these phenols cause skin, eyes, and respiratory system irritation. 4-cyanophenol is considered to be asphyxiant, which is a life-threatening situation. Also, 4-cyanophenol is toxic to fish [19]. 3-Nitrophenol is a priority pollutant as listed by the US EPA [20]. 3-Nitrophenol is toxic to eyes, skin, and the long exposure of this phenol may affect the body organs. Megharaj et al. [21] reported a decrease in the reproduction of the cyanobacteria at 2 µg/mL concentration of 4-nitrophenol. Besides, the authors also reported a decrease in carbohydrate production and carbon dioxide intake; leading to improper growth of the bacteria. The authors studied the effect of 3-nitrophenol at the molecular level and reported that phenol influenced the membrane properties and certain enzymes. Therefore, the analysis of these phenols is very important before supplying drinking water.

Before using any analytical technique, sample preparation is carried out to get rid of many other constituents [22]. Besides, the sample preparation is used to concentrate the analytes of interest as, sometimes, the analytes are at low concentrations and beyond the scope of the detection of the analytical instrument [23]. Various sample preparation methods are used for the analysis of phenols and these include liquid-liquid extraction (LLE), solid-phase extraction (SPE), etc. [24,25,26,27]. Presently, solid-phase extraction (SPE) is gaining importance in sample preparation because of its ease of use and wide range of applications. Additionally, it is also economic and green in nature as it uses a low amount of sorbent, reagents, and solvents. Many sorbents have been used in various sample preparation methods but nano-sorbents are gaining significance as SPE sorbents due to their wide range of sorption capabilities, high surface energy, and activities. Keeping all these facts into consideration, the multi-walled carbon nanotubes (MWCNTs) were synthesized [28] and used in SPE. The analytical technique selected in this study was capillary electrophoresis (CE). The choice of this technique is based on ease of operation, economic, and green in nature because it uses fewer solvents.

## 2. Materials and Methods

### 2.1. Chemicals and Reagents

Phenol, 4-cyanophenol, 3-nitrophenol, sodium dihydrogen phosphate, and sodium phosphate (AR grade) were acquired from Sigma-Aldrich Chemical Co., St. Louis, MO, USA. LiChrosolve methanol was supplied by Fisher Scientific (Fairlawn, New Jersey, USA). The phosphate buffer of 0.015 M (pH 7.0) was prepared by standard procedure. The solutions (10.0–100.0 µg/L) of phenols were prepared in phosphate buffer for CE experiments. For sample preparation studies, 10–60 mg/mL concentration solutions were prepared in deionized water.

### 2.2. Instruments Used

The CE was of Quanta 4000 acquired from Waters Chromatography, Millipore, Bedford, MA, USA and a Millennium 2000 software. The analysis was performed using a fused silica capillary (60 cm; with 75 μm id) and was acquired from Waters, Bedford, MA, USA and UV detector (214 nm). pHs of phenol solutions were fixed by a pH meter Orion Research Inc., Jacksonville, FL, USA. A 12-way standard SPE manifold operated with a vacuum pump AP-9950 from Auto Science (Shanghai, China) was employed for the SPE process.

### 2.3. Synthesis of MWCNTs

The multi-walled carbon nanotubes were prepared by Chemical Vapor deposition (CVD) method as reported earlier. The detail of the procedure is described elsewhere else [29].

### 2.4. Fabrication of SPE Cartridge

The MWCNTs (1.0 g) were weighed accurately and transferred to a plastic syringe of 5.0 cm length and 5.7 mm in diameter. The plastic syringe was purchased from a local market. The plastic syringe containing MWCNTs was vortexed for 15 min. The so obtained plastic syringe containing MWCNTs was known as a MWCNTs-based cartridge. The so obtained plastic syringe containing MWCNTs was washed with deionized water following methanol.

### 2.5. Capillary Electrophoretic Conditions

The CE equipment used is already described in Section 2.3. The experiments were carried out as described elsewhere [30] with at 18 kV voltage, 214 nm detection, and phosphate buffer (pH 7.0, 15 mM) as background electrolyte at 25 ± 1 °C temperature. The sampling was performed for 25 s through hydrostatic means of injection. The data was screened at 25 points/second. The electropherograms of the phenols in the standard solution and water samples were compared to find out the phenols in the water sample qualitatively. It was done with the help of migration times of phenols in the standard and water samples. The concentrations of phenols in water samples were determined by the following equation. The internal standard used was phenol in this study.

Conc._in water sample_ = [PAISK/PAISU] × [AU/AK] × [Conc._known_] (1)

where AISK = peak area of the internal standard in known, AISU = peak area of the internal standard in a water sample, AK = are of known and AU= area of a water sample.

### 2.6. Phenols Extraction From Water by SPE

1.0 mL volume of the different concentrations of both phenols i.e., 4-cyanophenol and 3-nitrophenol were added to 999 mL of deionized water to get concentrations of 10–60 mg/L, separately and respectively, vortexed and reserved for full night. Phenol was added as an internal standard at the concentration of 50 mg/L. These solutions were passed through the pre-conditioned MWCNTs cartridges at different flow rates (mL/min). After this, the deionized water was passed through the cartridges. The cartridges were air-dried. After this, the sorbed 4-cyanophenol and 3-nitrophenol were desorbed from MWCNTs using MeOH with TFA (0.05%) two times at different flow rates. These fractions were collected and evaporated to 0.10 mL in a nitrogen atmosphere under a vacuum. These volumes were used for the analysis of 4-cyanophenol and 3-nitrophenol by CE.

## 3. Results and Discussion

### 3.1. Preparation and Characterization of MWCNTs

The multi-walled carbon nanotubes (MWCNTs) were produced as an economic method. The characterization of multi-walled carbon nanotubes (MWCNTs) is previously designated and the attracted person can refer that work [28].

### 3.2. Capillary Electrophoresis of Phenols Method

**Figure 1:**
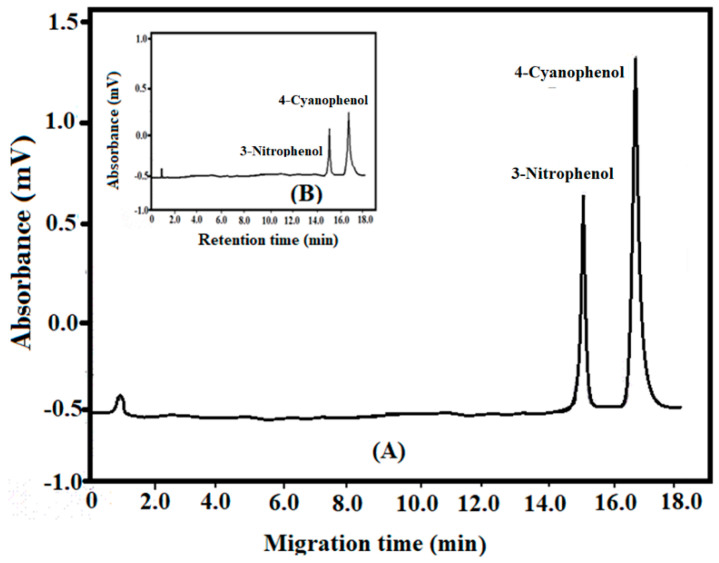
Electropherograms of 3-nitrophenol and 4-cyanophenol, (**A**): standard solution and (**B**): wastewater sample.

First of all, the best CE method was optimized by changing all the experimental parameters. The best experimental conditions were 18 kV voltage, 215 nm detection, and phosphate buffer (pH 7.0, 15 mM) as background electrolyte at 25 ± 1 °C temperature. The migration times of 3-nitrophenol and 4-cyanophenol were 15.05 and 16.50 min (Figure 1). The separation and resolution factors were 1.10 and 2.90. An analysis of Figure 1 indicates a clear cut separation of 3-nitrophenol and 4-cyanophenol. CE parameters optimization was done via changing in the background electrolyte composition and pH, applied voltage, temperature, the amount injected, and detection wavelength. The best separation parameters are described in this article. The determination of phenols (n = 6) in water was performed by recording the migration times and comparing them with the standards. These were identified by migration times and the amount in water sample was ascertained by quantitative analysis as mentioned above.

### 3.3. Capillary Electrophoresis Validation

The standard protocols were used to validate the CE procedure [30]. The parameters considered were accuracy, precision, specificity, linearity, limit of detection (LOD), limit of quantification (LOQ), and ruggedness. The accuracy of the method was determined by the quality controls of phenols at 10, 20, and 30 µg/L. The electrophoretic separation was performed six times (n = 6). Accuracy was established by interpolation of six replicates electropherogram areas of the phenols. These ranges showed a quite acceptable accuracy of the described method. The precision data was computed at three different concentrations of 10, 20, and 30 µg/L. Six sets of electrophoretic separations were carried out for all the concentrations. The %RSD, correlation coefficients, and confidence levels were 0.61, 0.9998, and 97.7 for nitrophenol while these values of 4-cyanophenol were 0.76, 0.9997 and 96.6 (Table 1). These results clearly showed good precision of the method. CE method is good enough specific as indicated by Figure 1. The migration times were almost similar in both standard and sample solutions. Furthermore, no change in migration time was seen when added some impurities in the solution confirming the specificity of the method. The linearity was performed by the electropherograms areas vs. concentrations; concentrations 10–50 µg/L. The plots were linear for these concentrations at n = 6. The linear regression analysis was done on the resultant plots. The LOD and LOQ were 0.1 and 1.0 and 0.32 and 3.5 µg (for 3-nitrophenol and 4-cyanophenol) with acceptable values of %RSD, correlation coefficients, and confidence levels (Table 1). The ruggedness was performed for intra- and inter-days assays with %RSD, correlation coefficients, and confidence levels 0.91–1.43, 0.9996–9998, and 96.5–96.5; showing ruggedness of the method.

### 3.4. SPE Phenols Extraction

The amounts of phenols, pH of phenol solution, contact time, and amount of MWCNTs were varied to get maximum uptake of phenols in water. The outcomes of these experiments are discussed in the below sub-sections.

#### 3.4.1. Phenols Concentrations

**Figure 2:**
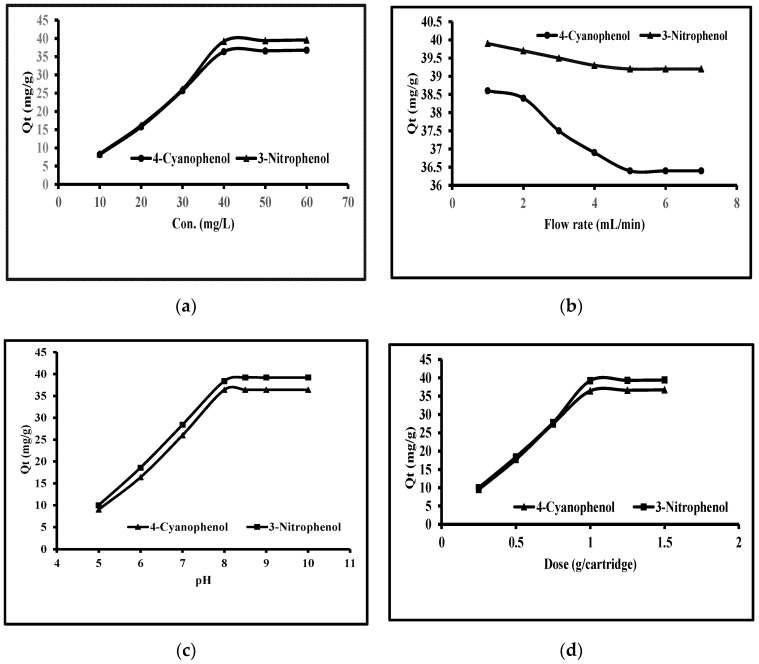
Effect of (**a**): conc., (**b**): flow rates, (**c**): pH, and (**d**): dose for the removal of phenols.

The effect of phenol concentration was performed from 10.0 to 60.0 mg L^−1^. The other conditions adjusted were pHs of 8.0 and 8.5 of water for 4-cyanophenol and 3-nitrophenol, a flow rate of water 5 mL/min, and the flow rate of eluting solvent 0.1 mL/min. This is shown in Figure 2a. The amounts up taken by MWCNTs were from 8.2 to 36.8 mg/g for 4-cyanophenol at 10 to 40 mg/L concentration. Furthermore, the augment of concentration to 60 mg/L could not increase the uptake of 4-cyanophenol. Consequently, 40 mg/L was the maximum concentration of 4-cyanophenol for the best uptake on MWCNTs. Similarly, the amounts up taken by MWCNTs were from 8.4 to 39.2 mg/mg for 3-nitrophenol at 10 to 40 mg/L concentration. Furthermore, the augment of concentration to 60 mg/L could not increase 3-nitrophenol uptake. Consequently, 40 mg/L was the best concentration for 3-nitrophenol maximal uptake on MWCNTs. Finally, it was supposed that the maximum phenols uptake was at 40 mg/L.

#### 3.4.2. Extraction Flow Rate

The effect of extraction time was measured in terms of the flow rates through SPE. The flow rates were varied from 1.0 to 7.0 mL/min and results are given in Figure 2b. The amounts extracted of 3-nitrophenol were 39.9, 39.7, 39.5, 39.3, 39.2 39.2m and 39.2 mg/g at 1.0, 2.0, 3.0, 4.0, 5.0, 6.0, and 7.0 L/min while these values were 38.6, 38.4, 37.5, 36.9, 36.4, 36.4 and 36.4 for 4-cyanophenol. It is clear from this data that the extraction of 3-nitrophenol was greater than 4-cyanophenol. The extraction values were greater at a low flow rate. It was observed that the time taken was high at 1.0 to 4.0 flow rates while the amount extracted was low at 6.0 and 7.0 mL/min flow rates. Consequently, 5.0 mL/min was considered to be the best one.

#### 3.4.3. pH Effect

The effect of phenol solutions pH was performed from 5 to 10 pHs. The other conditions fixed were as above (Figure 2c). The amounts taken up by MWCNTs were 9.0 to 36.4 mg/g for 4-cyanophenol at pH 5 to 8. Furthermore, augment of pH to 10 did not increase the uptake of 4-cyanophenol. Consequently, pH 8 was the best one for 4-cyanophenol maximum uptake on MWCNTs. Similarly, the amounts up taken by MWCNTs were from 10.0 to 39.2 mg/g for 3-nitrophenol at pH 5 to 8.5. Furthermore, augment of pH to 10 could not lead to an increase in 3-nitrophenol uptake. Consequently, pH 8.5 was thought to be the best for 3-nitrophenol maximum uptake on MWCNTs. Finally, it was supposed that the best pHs were 8.0 and 8.5 for 4-cyanophenol and 3-nitrophenol.

#### 3.4.4. MWCNTs Dose

The effect of MWCNTs dose was performed from 0.25 to 1.5 g per cartridge. The other conditions fixed were as mentioned above (Figure 2d). The amounts up taken by MWCNTs were from 9.5 to 36.4 mg/g for 4-cyanophenol at 0.25 to 1.0 g dosage of MWCNTs. Furthermore, augment of MWCNTs to 1.5 could not lead any uptake increase of 4-cyanophenol. Consequently, 1.0 g dose of MWCNTs was supposed to be the best for 4-cyanophenol maximum uptake on MWCNTs. Similarly, the amounts taken up by MWCNTs were from 10.0 to 39.2 mg/g for 3-nitrophenol at 0.25 to 1.0 g of MWCNTs. Furthermore, augment of MWCNTs to 1.5 g could not lead any uptake increase of 3-nitrophenol. Consequently, MWCNTs 1.0 g was supposed to be the best dosage of the MWCNTs for 3-nitrophenol maximum uptake.

#### 3.4.5. Desorption of Phenols

**Figure 3:**
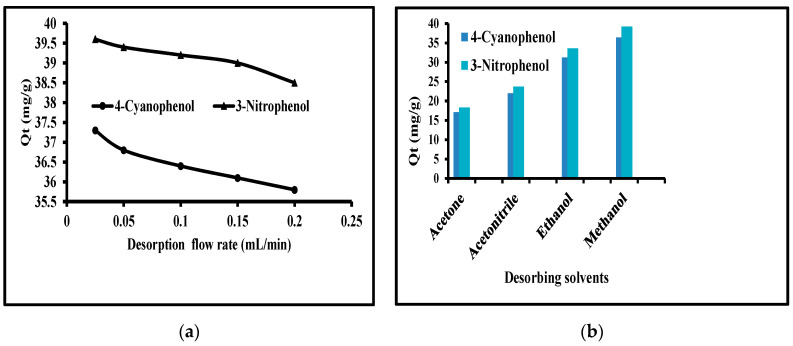
Effect of (**a**): flow rates of desorbing solvents and (**b**): different solvents from SPE cartridge.

The study of pH effect on the desorption of phenols from MWCNTs is one of the essential parts of this study. This was achieved by using different flow rates with different eluting solvents. The flow rates used were 0.025, 0.05, 0.1, 0.15, and 0.2 mL/min. The results of this set of studies are graphed in Figure 3a. A look of this figure shows that the values of desorption of 4-cyanophenol were 37.3, 36.8, 36.4, 36.1, and 35.8 mg/g at 0.025, 0.05, 0.1, 0.15, and 0.2 mL/min flow rates while at these flow rates the amount of desorption for 3-nitrophenol were 39.6, 39.4, 39.2, 39.0, and 38.5. It is clear from these values that maximum desorption has occurred at a low flow rate but it took a large time while at high flow rates the amounts of desorption were low. Consequently, 0.1 mL/min was considered to be the best one with 36. In addition, 39.2 mg/g desorption of 4-cyanophenol and 3-nitrophenol. Besides, the various desorbing eluents were organic solvents such as methanol, ethanol, acetonitrile, and acetone. The amount disrobed using these solvents (with 0.01% TFA) is given in Figure 3b. The amounts desorb were in the order of acetone (17.1 mg/g) < acetonitrile (22.0 mg/g) < ethanol (31.2 mg/g) < methanol (36.4 mg/g) for 4-cyanophenol. The same order was observed for 3-nitrophenol with 18.3, 23.7, 33.6, and 39.2 mg/g amounts desorbed. This order was because of the different polarities of these solvents. It is clear that the maximum desorption of the reported phenols was with methanol. Furthermore, the desorption was optimized by adding some acids such as acetic acid, trifluoroacetic acid, hydrochloric acid, and sulfuric acid. Among these, trifluoroacetic acid gave the best results with 0.01% concentration in methanol. Under these conditions, the maximum desorption was 91% and 98% for 4-cyanophenol and 3-nitrophenol. To compare the results of MWCNTs as new sorbent, the experiments were also carried out with a commercially available C_18_ cartridge. The experiments were carried out under identical and optimized conditions. The maximum recoveries of 4-cyanophenol and 3-nitrophenol were 84.5 and 88.6%. These results clearly indicated that MWCNTs material is better as the new sorbent in SPE.

## 4. Application of SPE and CE Methods in Spiked Real Water Samples

The municipal wastewater samples (1 L; 5.6 pH) were collected from New Delhi, India and centrifuged to remove any solid particle. These were analyzed by CE for identifying the presence of any phenol. The water sample was spiked 4-cyanophenol and 3-nitrophenol to get a concentration of 40 mg/L. This sample was kept overnight. This was passed through SPE cartridge at 5 mL/min flow rate. The phenols were desorbed by MeOH (0.1% trifluoroacetic acid) at 0.1 mL/min flow rate. The eluent was concentrated and analyzed by CE as described above. It was observed that no other peak than 4-cyanophenol and 3-nitrophenol was appeared in the electropherogram (Figure 1) confirming the specificity of SPE and CE methods. The percentage recoveries were 91 and 98% of cyano-phenol and 3-nitrophenol. Therefore, the SPE and CE methods may be used to analyze 4-cyanophenol and 3-nitrophenol in wastewater.

## 5. Conclusions

The SPE and CE methods were optimized and the best conditions were 40 mg/L concentration, 1.0 g MWCNTs per SPE cartridge, 5.0 mL/min flow rate of phenol containing water, 0.1 mL flow rate of eluting solvent for 4-cyanophenol and 3-nitrophenol. The maximum recoveries were 91% and 98% at 0.1 mL/min flow rate. The capillary electrophoresis was with 18 kV voltage, 214 nm detection, and phosphate buffer (pH 7.0, 15 mM) as background electrolyte at 25 ± 1 °C temperature with 15.05 and 16.5 min migration times. The separation and resolution factors were 1.10 and 2.90. The LOD and LOQ were in the range of 0.1–1.0 and 0.32–3.5 µg. These methods were applied successfully for the extraction and estimation of 4-cyanophenol and 3-nitrophenol in the municipal wastewater. The reported methods are reproducible, efficient, and practical for the estimation of these phenols in water. Finally, the reported methods are reproducible, efficient, and practical for the estimation of these phenols in water and may be used for the analysis of these phenols in any water resource.

## Figures and Tables

**Table 1:** Validation parameters of capillary electrophoresis.

Sl. No.	Validated Parameters	3-Nitrophenol			4-Cyanophenol		
		% RSD	Correlation Coefficients	Confidence Levels	% RSD	Correlation Coefficients	Confidence Levels
1.	Precision	0.61	0.9998	97.7	0.76	0.9997	96.6
2.	Linearity	0.56	0.9998	97.5	1.21	0.9997	96.5
3.	LOD	0.84	0.9998	96.6	1.31	0.9996	95.8
4.	LOQ	0.84	0.9997	96.5	1.06	0.9996	96.9
5.	Ruggedness	0.91	0.9998	96.7	1.43	0.9996	96.5
